# Surfactant Protein A Inhibits Human Rhinovirus C Binding and Infection of Airway Epithelial Cells from Pediatric Asthma

**DOI:** 10.3390/v16111709

**Published:** 2024-10-30

**Authors:** Sasipa Tanyaratsrisakul, Yury A. Bochkov, Vanessa White, Heejung Lee, Jessica Loeffler, Jamie Everman, Allison M. Schiltz, Kristy L. Freeman, Katharine L. Hamlington, Elizabeth A. Secor, Nathan D. Jackson, Hong Wei Chu, Andrew H. Liu, Julie G. Ledford, Monica Kraft, Max A. Seibold, Dennis R. Voelker, Mari Numata

**Affiliations:** 1 Department of Medicine, National Jewish Health, Denver, CO 80206, USA; sasipat@arizona.edu (S.T.); whitev@njhealth.org (V.W.); heejung.lee@takeda.com (H.L.); jessjloeffler@gmail.com (J.L.); chuh@njhealth.org (H.W.C.); voelkerdr50@gmail.com (D.R.V.); 2 Asthma and Airway Disease Research Center, University of Arizona, Tucson, AZ 85724, USA; jledford@arizona.edu; 3 Department of Pediatrics, University of Wisconsin School of Medicine and Public Health, Madison, WI 53792, USA; yab@medicine.wisc.edu; 4 Center for Genes, Environment and Health, National Jewish Health, Denver, CO 80206, USA; evermanj@njhealth.org (J.E.); secore@njhealth.org (E.A.S.); jacksonna@njhealth.org (N.D.J.); seiboldm@njhealth.org (M.A.S.); 5 Section of Pediatric Pulmonary & Sleep Medicine, Children’s Hospital Colorado and University of Colorado School of Medicine, Aurora, CO 80045, USA; allison.schiltz@childrenscolorado.org (A.M.S.); kate.h.smith@cuanschutz.edu (K.L.H.); andrew.liu@childrenscolorado.org (A.H.L.); 6 Department of Cellular and Molecular Medicine, University of Arizona, Tucson, AZ 85719, USA; 7 Department of Medicine, Icahn School of Medicine at Mount Sinai, New York, NY 10029, USA; monica.kraft@mssm.edu; 8 Department of Pediatrics, National Jewish Health, Denver, CO 80206, USA; 9 Division of Pulmonary Sciences and Critical Care Medicine, Department of Medicine, University of Colorado-AMC, Aurora, CO 80045, USA

**Keywords:** pulmonary surfactant protein A, human rhinovirus C, pediatric asthma, innate immunity

## Abstract

Rhinovirus C (RV-C) infection can trigger asthma exacerbations in children and adults, and RV-C-induced wheezing illnesses in preschool children correlate with the development of childhood asthma. Surfactant protein A (SP-A) plays a critical role in regulating pulmonary innate immunity by binding to numerous respiratory pathogens. Mature SP-A consists of multiple isoforms that form the hetero-oligomers of SP-A1 and SP-A2, organized in 18-mers. In this report, we examined the efficacy of SP-A to antagonize RV-C infection using the wild-type (RV-C15) and reporter-expressing (RV-C15-GFP) viruses in differentiated nasal epithelial cells (NECs) from asthmatic and non-asthmatic children. We also determined the antiviral mechanism of action of SP-A on RV-C15 infection. The native SP-A was purified from alveolar proteinosis patients. The recombinant (r) SP-A1 and SP-A2 variants were expressed in FreeStyle™ 293-F cells. SP-A reduced the fluorescent focus-forming units (FFUs) after RV-C15-GFP infection of NECs by 99%. Both simultaneous and 4 h post-infection treatment with SP-A inhibited RV-C15 and RV-C15-GFP viral RNA load by 97%. In addition, the antiviral genes and chemokines (IFN-λ, IRF-7, MDA-5, and CXLC11) were not induced in the infected NECs due to the inhibition of RV-C propagation by SP-A. Furthermore, SP-A bound strongly to RV-C15 in a dose- and Ca^2+^-dependent manner, and this interaction inhibited RV-C15 binding to NECs. In contrast, rSP-A1 did not bind to solid-phase RV-C15, whereas the rSP-A2 variants, [A_91_, K_223_] and [P_91_, Q_223_], had strong binding affinities to RV-C15, similar to native SP-A. This study demonstrates that SP-A might have potential as an antiviral for RV infection and RV-induced asthma exacerbations.

## 1. Introduction

Rhinovirus (RV) infection frequently causes wheezing in pediatric patients and increases the risk for developing asthma [1]. While usually presented as a mild infection in healthy individuals, rhinovirus infection in individuals with chronic lung diseases can be a major trigger for life-threatening airway obstruction, wheezing illnesses, as well as asthma and COPD exacerbations [1,2,3].

Among the three species of RV (A, B, and C), rhinovirus C (RV-C) is more frequently associated with wheezing illnesses and exacerbations of asthma in young children [4,5,6,7,8]. The cellular receptor for RV-C is cadherin-related family member 3 (CDHR3), for which expression is restricted to the ciliated airway epithelial cells [9,10,11]. For this reason, submerged cultures of primary epithelial cells and continuous cell lines are not susceptible to RV-C infection [12,13]. Fully differentiated airway epithelial cells cultured at the air–liquid interface (ALI) are permissive to RV-C infection and provide a model for investigating the mechanisms of infection and therapeutic interventions in vitro [12,13].

Pulmonary surfactant protein A (SP-A) is a calcium-dependent (C-type) lectin synthesized by alveolar type II cells and epithelial club cells [14]. There are two genes encoding for human SP-A, SP-A1, and SP-A2. Mature SP-A comprises 18 monomers in combination of both SP-A1 and SP-A2, forming a bouquet-like structure [15]. The SP-A1 variants contain V or L amino acid residues at position 50 and R or W at position 219 [V/L_50_, R/W_219_]; and the SPA-2 variants contain P or A at position 91 and Q or K at position 223 [p/A_91_, Q/K_223_]. SP-As are important molecules for host defense in the airways through their anti-infectious and immunomodulatory roles [16,17]. Moreover, SP-As inhibit respiratory viral infections with influenza A virus (IAV), respiratory syncytial virus, and coronavirus through binding to those viruses and inhibiting viral attachment to the host cells [18,19,20,21,22,23]. We previously reported that SP-A could reduce the infection of HeLa and BEAS2B cells with a major receptor group RV-A16 by inhibiting its attachment to host cells [24]. In this report, we determined whether SP-A could inhibit RV-C infection using differentiated primary human nasal epithelial cells (NECs) and examined the mechanisms underlying the antiviral actions of SP-A against RV-C15 infection.

## 2. Materials and Methods

### 2.1. Human Nasal Epithelial Cell (NEC) Culture

Primary human NECs, obtained from 10 asthmatic and 6 non-asthmatic donors, were randomly selected from deidentified samples that were collected and banked as part of the Denver Asthma Panel Study (DAPS). The National Jewish Health Institutional Review Board approved the research on nasal cells (approval number COMIRB 15-0138 and COMIRB 17-1416). All participants and their guardians gave written informed assent and consent, respectively. Symptom severity was determined using the Composite Asthma Severity Index (CASI) score ranging from 0 to 20 points, with higher scores indicating higher levels of disease severity [25,26]. Genotyping of CDHR3 (cadherin-related family member 3) rs6967330 locus was performed using the TaqMan^TM^ SNP assay (Thermo Fisher, Waltham, MA, USA, assay ID: C_29286131_10) as previously described [10]. Participants’ demographic and clinical information are listed in Table 1.

NECs were grown and differentiated at the air–liquid interface (ALI) as previously described [27]. Briefly, the NECs were expanded on irradiated fibroblasts for 3–5 days. Then, the cells were seeded at 2 × 10^4^ cells/well on 6.5 mm collagen-coated transwell inserts (Corning, Kennebunk, ME, USA) using PneumaCult Ex plus media (StemCell Technologies, Vancouver, BC, Canada). Once the cultures reached confluence, the apical media was removed, and the basolateral media was changed to PneumaCult ALI media (StemCell Technologies). Cells were differentiated to mature mucociliary epithelium for 21–24 days before infection.

### 2.2. Virus Production and Infection

The recombinant wild-type RV-C15 and the RV-C15 variant expressing green fluorescent protein (RV-C15-GFP) were generated by a reverse genetics system [11,28] as previously published but with modification. Briefly, the viral RNA synthesized from linearized pC15 or pC15-GFP cDNA was transfected into HeLa-E8 cells, expressing CDHR3, using lipofectamine 2000 (Thermo Fisher). At 24 h post transfection, the cells were lysed by three freeze and thaw cycles, followed by an RNase A treatment. Virus particles were isolated from the clarified cell lysate by pelleting through a 30% sucrose cushion at 40,000 rpm. The pellets were then resuspended in PBS with 0.01% BSA and stored at −80 °C. The viral titer was quantified by RT-qPCR and expressed as viral RNA copy number per µL.

At 24 h prior to infection, the apical side of differentiated NECs was washed with Dulbecco’s phosphate-buffered saline (DPBS, Thermo Fisher), and the growth medium was replaced. On the next day, the basolateral sides were washed with DPBS twice. Then, RV-C15 or RV-C15-GFP at 2 × 10^4^ copy numbers/well in 75 µL of media with or without SP-A was added apically, and the growth medium was replaced in the basal compartment. After a 4 h incubation at 34 °C, the cells were washed with DPBS to remove unbound viruses followed by adding 75 µL of media with or without SP-A apically, and 500 µL of media alone in basolateral chamber. For the post-infection treatment, SP-A was added apically after viral challenge at 4 h or 24 h post infection, and the cells were incubated at 34 °C for 24–48 h.

### 2.3. SP-A Preparation

Native SP-A was purified from the bronchoalveolar lavage (BAL) from an alveolar proteinosis patient as described [14] but with modification. Briefly, lipids were extracted using 98% butanol and dried under a gentle stream of N_2_. Then, the protein pellet was dissolved and dialyzed in 5 mM Tris-HCl buffer. The soluble SP-A was purified using a mannose–sepharose 6B affinity column in the presence of 5 mM calcium, and subsequently eluted with buffer containing 7 mM EDTA. EDTA was removed by dialysis with 5 mM Tris-HCl pH7.4. Recombinant SP-A isoforms were expressed in 293F expression system (Thermo Fisher) according to the manufacturer protocol. Briefly, the cells were transfected with 30 µg of plasmid using 293 fectin for 48 h. Culture media harboring soluble recombinant SP-As were purified by mannose column, as described above. The purified proteins were analyzed by agarose gel electrophoresis. The molecular weight of native and recombinant SP-As was confirmed by size exclusion chromatography using a Superdex200 10/300 GL (Cytiva, Marlborough, MA, USA) column. The protein concentration was determined by a spectrophotometer at A_280_ nm and calculated using the specific molar absorption coefficient.

### 2.4. Quantification of Virus Infection and Antiviral Responses

At 24 h after infection, fluorescent and differential contrast images were obtained using an inverted fluorescence microscope. The cells infected with RV-C15-GFP express GFP and were visualized by fluorescence microscopy as fluorescent focus-forming units (FFUs). FFU numbers induced by RV-C15-GFP in NECs in ALI cultures were measured using SlideBook software Version 6, and 6 non-overlapped images from each well were averaged. Cellular RNA was extracted from NECs at 4 h, and 48 h, post-infection using the RNeasy mini kit (Qiagen, Hilden, Germany), then the cDNA was synthesized using SuperscriptIII and random primers (Thermo Fisher). For quantification of viral mRNA, a 388 bp fragment of viral cDNA, amplified using primer F 5′-GCACTTCTGTTTCCCCGG-3′ and R 5′-CGGACACCCAAAGTAGTCGG-3′ was produced. The viral cDNA fragment was gel-purified, and the concentration was determined by A_260nm_. The molecular weight of the cDNA fragment was calculated from MW = ((A + T) × 617.4) + ((C + G) × 618.4) g/mol, where A, T, C, and G were the number of nucleotides A, T, C, and G in cDNA (+strand), respectively. Then, the copy number of cDNA was calculated by the copy number = 6.022 × 10^23^ molecules/mol × moles of cDNA × 2 (accounting for double-stranded cDNA). The cDNA was used as a template for RT-qPCR standard curve. The RT-qPCR was carried out using SYBR green master mix (Applied Biosystems, Waltham, MA, USA) or TaqMan master mix (Applied Biosystems), along with the primers listed in Table S1. The amplification was performed on the QuantStudio 3 (Thermo Fisher) real-time PCR system, in duplicate. Target genes include melanoma differentiation-associated protein 5 (*MDA5*), interferon regulatory factor 7 (*IRF7*), interferon lambda (*IFN*λ), and C-X-C motif chemokine 11 (*CXCL11*). The cycle threshold (Ct) of the target gene was normalized with those of a housekeeping gene, beta-actin or beta-glucuronidase (*GUSB*). The levels of secreted CXCL11 in the apical media were quantified by the human CXCL11 ELISA kit (Peprotech, Cranbury, NJ, USA).

### 2.5. RV-C15 and SP-A Solid Phase Binding Assay

First, a 96-well, half-area plate (Thermo Fisher) was coated with RV-C15 (10^4^ copy numbers/well) in PBS at 4 °C overnight [29]. After blocking with 3% BSA in 20 mM Tris, 150 mM NaCl, SP-A diluted in the blocking buffer was added and incubated for 1 h. The interaction was determined in the presence of 5 mM EDTA or 5 mM CaCl_2_. Bound SP-A were detected by the anti-human SP-A [30] conjugated with HRP. After adding OPD substrate and stop solution, the absorbance at 490 nm was measured.

### 2.6. RV-C15 Attachment to the Differentiated Mucociliary NECs

Apical chambers of differentiated NECs were washed with DPBS twice, and fresh media was replaced in the basolateral chamber. Cultures were then infected with RV-C15 (2 × 10^4^ copy numbers/well) in apical media containing SP-A at 0 to 400 µg/mL. NECs were incubated at 4 °C for 4 h to allow for binding but to prevent viral endocytosis. After incubation, the cells were washed 3 times with DPBS to remove the unbound virus; the cultures were then lysed for RNA extraction and RT-qPCR.

### 2.7. Statistical Analysis

The data were analyzed using the GraphPad Prism 9 software. For the native SP-A and SP-A isoforms inhibition of viral infection experiments (FFU numbers/well and RT-qPCR data) and antiviral gene expression data, the differences between the groups were analyzed by two-way ANOVA with Tukey’s multiple comparison test. For SP-A’s post-infection treatment and the binding of isoforms to RV-C15 experiments, data were analyzed by ANOVA with Tukey’s multiple comparison test. For the binding of SP-A and RV-C15, nonlinear regressions of total binding were used. For the SP-A inhibition of RV-C15 attachment to the cells, nonlinear regression in response to inhibitor concentrations were analyzed.

## 3. Results

### 3.1. SP-A Reduces RV-C15-GFP Infection in Human NECs

**Figure 1:**
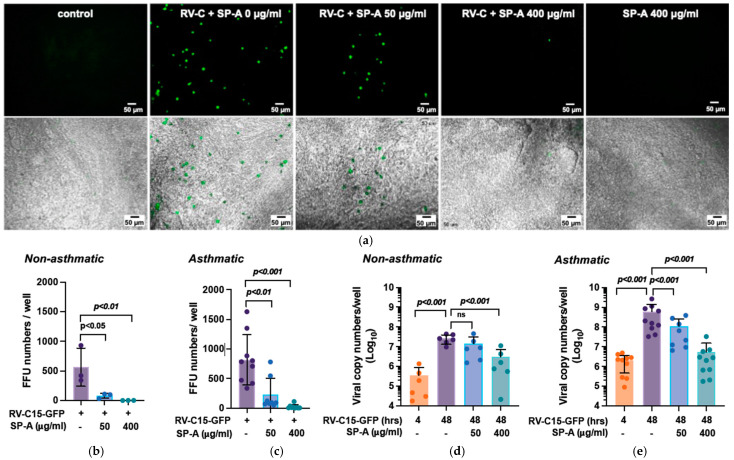
Native SP-A reduces RV-C15-GFP infection in human NECs. Differentiated NECs were incubated with RV-C15-GFP for 4 h with or without SP-A at 50 µg/mL or 400 µg/mL followed by washing with DPBS and replacing medium with or without SP-A. At 24 h after viral challenge, the cultures were visualized by fluorescence microscopy ((**a**), **upper** row panel), or combined fluorescence and differential interference contrast (DIC) ((**a**), **lower** row panel). The scale bar indicates 50 μm. Quantitative data of fluorescent focus-forming units (FFUs; numbers/well) in cells from the non-asthmatic (*n* = 3) (**b**) and asthmatic subjects (*n* = 9) (**c**) are shown. Each experiment was performed in duplicate. Data are means ± SD from six fields taken in each well. RV-C15-GFP viral copy numbers were measured by RT-qPCR at 48 h p.i. using NECs from (**d**) non-asthmatic (*n* = 6), and (**e**) asthmatic (*n* = 10) subjects. Data are shown as means ± SD from two to three individual experiments per donor.

To examine whether SP-A inhibits RV-C15 infection, differentiated NECs cultured at ALI were infected with reporter-expressing RV-C15-GFP (2 × 10^4^ viral copy numbers/well) in the presence or absence of SP-A. Fluorescence microscopy was used to visualize GFP expression from RV-C15-GFP-infected cells (Figure 1a, upper panels). At 24 h post-infection, the fluorescent focus-forming unit (FFU) numbers were reduced by SP-A in both the non-asthmatic and asthmatic cells in a dose-dependent manner (Figure 1a). FFU numbers in the RV-C15-GFP-infected non-asthmatic NECs were 564.4 ± 320.4 FFU numbers/well (Figure 1b); SP-A treatment reduced the foci counts to 79.8 ± 42.3 FFU numbers/well (14.1%) and 2.1 ± 1.4 FFU numbers/well (0.3%), with simultaneous treatments with SP-A at 50 and 400 µg/mL, respectively. The FFU numbers in asthmatic NECs with RV-C15-GFP alone were 819.7 ± 428.6 FFU numbers/well (Figure 1c). SP-A treatment reduced FFU numbers to 228.6 ± 275.8 FFU numbers/well (27.8%) at 50 μg/mL and 21.6 ± 35.2 FFU numbers/well (2.6%) at 400 μg/mL, respectively. As shown in Figure 1a, SP-A treatment alone (SP-A 400 μg/mL) did not cause any visible cytopathic effects in comparison to medium alone (control).

We next aimed to determine the effect of SP-A treatment at the viral genome level by performing RT-qPCR at 4 h and 48 h post infection (p.i.). Compared to an average of 3.5 ± 5.3 × 10^5^ viral copy numbers/well of cell-associated input for RV-C15-GFP at 4 h, the viral copy numbers increased by 2 logs at 48 h in non-asthmatic NECs (2.7 ± 1.3 × 10^7^ copy numbers/well) (Figure 1d). SP-A treatment at 50 µg/mL reduced viral copy number to 1.4 ± 1.7 × 10^7^ copy numbers/well (47.0% reduction) and SP-A at 400 µg/mL reduced the number to 3.1 ± 4.3 × 10^6^ copy numbers/well (88.4% reduction). NECs from asthmatic subjects had approximately 10-fold higher cell-associated viral genome numbers at 4 h compared to non-asthmatics (Figure 1d,e). The viral loads in asthmatic NECs were 2.1 ± 1.6 × 10^6^ copy numbers/well at 4 h and 5.8 ± 8.0 × 10^8^ copy numbers/well at 48 h p.i. (Figure 1e). SP-A treatment reduced the viral copy numbers by 80.5% (1.1 ± 1.4 × 10^8^ copy numbers/well at 50 µg/mL of SP-A) and 99.1% (5.5 ± 9.9 × 10^6^ copy numbers/well at 400 µg/mL of SP-A).

### 3.2. SP-A Reduces RV-C15 Propagation 4 H After Viral Infection Has Been Established

**Figure 2:**
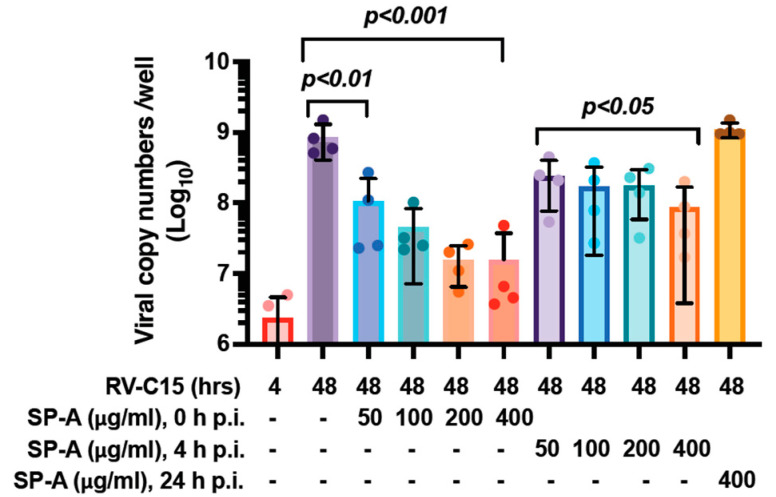
Native SP-A treatment at 4 h after viral challenge reduced viral copy numbers in a dose-dependent manner. Differentiated NECs were incubated with RV-C15 at 2 × 10^4^ viral copy numbers/well for 4 h, then the unbound virus was removed by washing with DPBS. Native SP-A treatment was started at 0 h, 4 h, or 24 h p.i. of viral challenge at indicated concentration (50, 100, 200, or 400 µg/mL). Viral RNA was quantitated at 4 h or 48 h p.i. by RT-qPCR. Data were means ± SD from 4 non-asthmatic donors. *p*-values were shown in comparison to RV-C15 48 h alone.

We next sought to determine the inhibitory effect of SP-A after the virus has entered the cells and initiated protein translation and/or replication. Since SP-A efficiently inhibited virus infection in cells from both asthmatic and non-asthmatic donors, the cells from more readily available non-asthmatic subjects were used. SP-A was added to the NEC cultures at 0 h, 4 h, or 24 h after viral challenge. Viral copy numbers increased 300-fold from 4 h to 48 h p.i. (Figure 2). SP-A treatment at the time of viral exposure significantly reduced viral genome numbers by 7.8-, 19.1-, 53.8-, and 53.8-fold at 50, 100, 200, and 400 µg/mL, respectively, in a dose-dependent manner. The SP-A treatment at 4 h after viral inoculation also reduced RV-C15 viral copy numbers by 3.5-, 5.1-, 4.8-, and 10-fold at 50, 100, 200, and 400 µg/mL. However, SP-A treatment at 24 h p.i. failed to alter RV-C15 viral copy numbers even at the highest concentration.

### 3.3. SP-A Attenuates Antiviral Gene Expression and Chemokine Secretion Induced by RV-C15 Infection in NECs from Non-Asthmatics and Asthmatics

**Figure 3:**
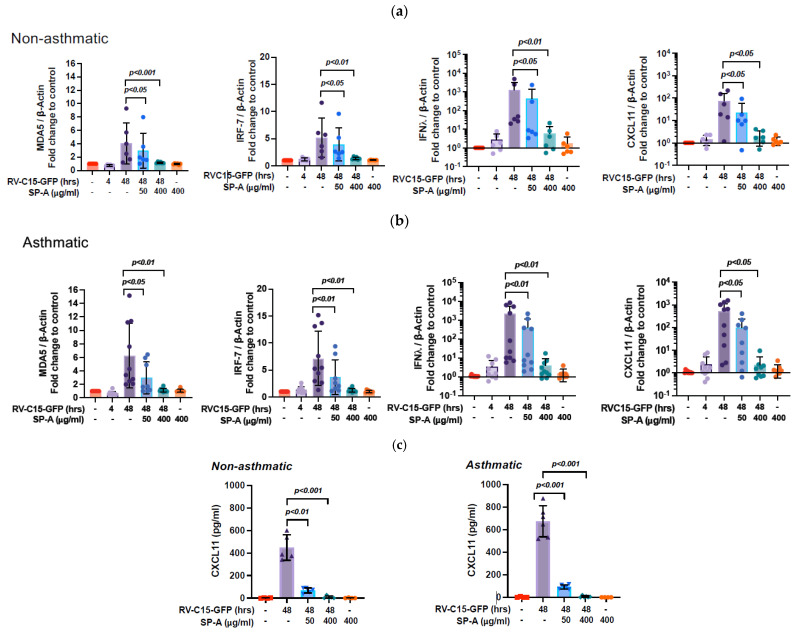
SP-A attenuates antiviral gene expression and chemokine secretion induced by RV-C15. Differentiated NECs were challenged with RV-C15-GFP as described in Figure 1. mRNA expression of *MDA5*, *IRF7*, *IFNλ*, and *CXCL11* were quantified by RT-qPCR by using RNA of NECs from non-asthmatics (*n* = 6) (**a**) and asthmatics (*n* = 10) (**b**). Data are shown as means ± SD from duplicated well in each condition for each donor. Secretion of CXCL11 in apical media was quantified by ELISA in (**c**). The data are means ± SD using cells from 5 non-asthmatic and 6 asthmatic donors; experiments limited by cell availability. *p*-values were shown in comparison to the condition of RV-C15 48 h alone.

Next, we determined the effects of SP-A treatment on RV-C15-GFP-induced host antiviral responses by RT-qPCR. We quantitated the expressions of melanoma differentiation-associated protein 5 (*MDA5*), interferon regulatory factor 7 (*IRF7*), type III interferon (*IFNλ*), and C-X-C Motif Chemokine Ligand 11 (*CXCL11*) (Figure 3) using the previously published methods [31]. The results showed that, at 48 h p.i., RV-C15-GFP infection induced the expression of these genes. RV-C15-GFP induced MDA5, IRF7, IFNλ, and CXCL11 transcription with 4.1-, 5.2-, 1241.0-, and 75.9-fold increases, respectively, in comparison to the sham infected condition in NECs from non-asthmatics (Figure 3a), whereas, in asthmatic NECs, RV-C15-GFP increased these genes 6.2-, 7.2-, 2252.3-, 531.2-fold, respectively (Figure 3b). The antiviral gene transcription decreased in the presence of SP-A to a level similar to the sham infected condition due to inhibition of RV-C15-GFP infection. In both non-asthma and asthma groups, simultaneous SP-A treatment significantly reduced antiviral genes and CXCL11 mRNA expressions in a dose dependent manner (Figure 3a,b). The secreted chemokine CXCL11 protein in apical media was measured by ELISA (Figure 3c). The RV-C15-GFP-induced CXCL11 secretion was 449.2 ± 114.2 pg/mL (non-asthmatic) and 675.4 ± 137.1 pg/mL (asthmatic) (Figure 3c). The RV-C15-GFP-induced CXCL11 secretion was markedly attenuated in the presence of SP-A.

### 3.4. SP-A Directly Binds to RV-C15 and Reduces Viral Attachment to NECs

**Figure 4:**
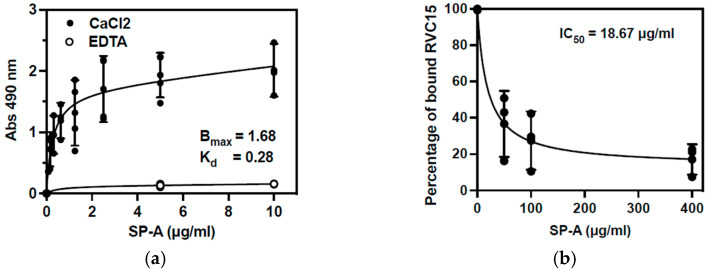
SP-A directly binds to RV-C15 and prevents its attachment to differentiated NECs in a dose-dependent manner. SP-A (0 to 10 µg/mL) was incubated for 1 h in RV-C15-precoated microtiter plate (**a**). The binding of SP-A and RV-C15 was quantified by anti-human SP-A HRP-conjugated antibody [27,30] in the presence of 5 mM EDTA (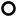) or 5 mM CaCl_2_ (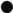). The maximum binding (B_max_) and dissociation constant (K_d_) determined by nonlinear regression analysis are indicated. (**b**) RV-C15 attachment to NECs was examined in the presence of various concentrations of SP-A (0 to 400 µg/mL) using cells from 4 asthmatic participants. Bound RV-C15 was quantified by RT-qPCR and results are shown as a percentage of viral binding compared to control. The concentration of SP-A that could inhibit the binding by 50% (IC_50_) is shown. The results represent means ± SD from 3 to 5 independent experiments.

To investigate how SP-A inhibits RV-C15 infection, we examined whether SP-A directly interacts with RV-C15. Various concentrations of SP-A (0–10 µg/mL) were incubated in microtiter plates precoated with RV-C15 (Figure 4a). SP-A binding was detected in the presence of 5 mM CaCl_2_ in a dose dependent manner, but it was not detected in the presence of 5 mM EDTA. The minute amount of non-specific binding of SP-A to the non-coated well, in the presence of CaCl_2_, (Figure S1) was subtracted from the total absorbance. Regression analysis showed the maximum binding (Bmax) of 1.68 at A_490nm_ and the dissociation constant (K_D_) of 0.28 µg/mL. We next examined whether SP-A inhibited RV-C15 attachment to the nasal airway epithelium (Figure 4b). The presence of SP-A reduced RV-C15 attachment to the differentiated NECs in a concentration-dependent manner with an IC_50_ of 18.67 µg/mL. SP-A at 400 µg/mL reduced RV-C15 attachment by 83%.

### 3.5. The Amino Acids at Positions 91 and 223 of SP-A2 Are Important for RV-C15 Binding

**Figure 5:**
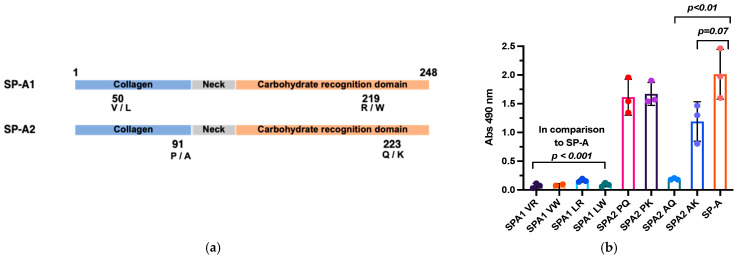
Amino acids at positions 91 and 223 determine the binding affinity of SP-A2 to RV-C15. The schematic (**a**) illustrates variation at amino acid positions of SP-A1 and SP-A2. (**b**) The binding affinity of native SPA or recombinant SP-A variants at 10 µg/mL to the solid phase RV-C15 in the presence of 5 mM CaCl_2_. The data are means ± SD from 3 independent experiments. *p*-values indicate samples that are significantly different from the native SP-A.

There are two genes encoding SP-A in humans (Figure 5a), namely SP-A1 and SP-A2. The coding region of SP-A1 has amino acid variations at positions 50 (V or L) and 219 (R or W), while SP-A2 has variations at positions 91 (P or A) and 223 (Q or K). To determine the effects of SP-A amino acid variations on RV-C15 binding, we generated eight recombinant (r) SP-A variants to test in the RV-C15 binding assay. A direct binding assay using 10 µg/mL of rSP-A and native SP-A (Figure 5b) showed low levels of rSP-A1 binding to RV-C15 (A_490nm_ of 0.06–0.16). However, the rSP-A2 [P_91_, Q_223_] and [P_91_, K_223_] variants exhibited strong binding to RV-C15 comparable to that of native SP-A (A_490nm_ of 1.61 and 1.67 vs. 2.01, respectively). Interestingly, rSP-A2 [A_91_, K_223_] binding was lower (A_490nm_ of 1.19) than that of native SP-A, and rSP-A2 [A_91_, Q_223_] showed very low binding to RV-C15 (A_490nm_ of 0.19).

### 3.6. The Amino Acids at Positions 91 and 223 of SP-A2 Are Important for Inhibition of RV-C15 Infection

**Figure 6:**
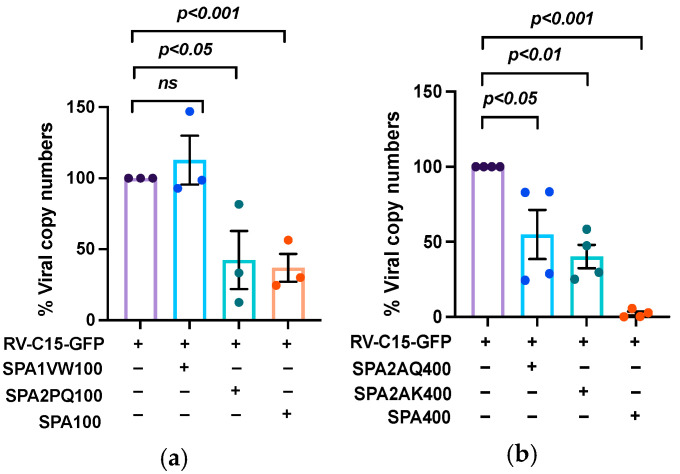
The SP-A inhibition of RV-C15-GFP infection corresponds to SP-A virus binding efficiency. (**a**) Differentiated NECs were infected for 48 h with RV-C15-GFP in the presence or absence of native SP-A, or recombinant SP-A1 [V_50_, W_219_] (SPA1VW) or SP-A2 [P_91_, Q_223_] (SPA2PQ) at 100 µg/mL. The results are shown in comparison to RV-C15-GFP control (expressed as 100%). (**b**) The cultures were infected as in panel A and treated with native SP-A, SP-A2 [A_91_, Q_223_], or SP-A2 [A_91_, K_223_] at 400 µg/mL. The data derived from cells obtained from 3 to 4 non-asthmatic participants. The data are means ± SD. *p*-values indicate samples that are significantly different from the RV-C15 GFP infection control.

Next, we tested the ability of rSP-A1 and rSP-A2 to inhibit RV-C15 infection. Differentiated NECs were infected with RV-C15-GFP in the presence of native SP-A, rSP-A1 [V_50_, W_219_], or rSP-A2 [P_91_, Q_223_] at 100 µg/mL. In agreement with the RV-C15 binding results, the presence of rSP-A1 did not inhibit RV-C15-GFP infection (112.8 ± 29.8% viral copy numbers/well) compared to RV-C15-GFP alone, which is shown as 100% (Figure 6a). In contrast, rSP-A2 [P_91_, Q_223_] reduced the viral progeny yields similarly to native SP-A (42.4 ± 35.4% and 36.9 ± 17.0%, respectively). The inhibitory effects of the rSP-A2 variants, [A_91_, Q_223_] and [A_91_, K_223_], that had weaker binding to RV-C15 were examined at 400 µg/mL (Figure 6b). Compared to RV-C15-GFP infection control, rSP-A2 [A_91_, Q_223_] and [A_91_, K_223_] reduced the percentage of viral copy numbers to 54.8 ± 32.7 and 40.2 ± 15.5%, respectively. Furthermore, inhibition by SP-A at 400 µg/mL reduced the viral RNA copies to 2.2 ± 2.6% of RV-C15-GFP alone. Unfortunately, we had technical difficulties with obtaining high concentrations of some recombinant SP-A1 and SP-A2 isoforms. Because of this reason, we were unable to use 400 μg/mL for all of the experiments.

## 4. Discussion

RV-C infection induces life-threatening exacerbations in patients with asthma and chronic lung diseases [4,5,7,8,9,32]. Asthma exacerbations caused by RV-C are a major cause of childhood hospitalization and represent a large healthcare expense [3]. However, due to a large number of circulating virus types and a lack of cross-protection among them, vaccine or effective treatment options for RV infections are currently not available [2,6]. Human surfactant protein A (SP-A), a pulmonary collectin, directly interacts with allergens, pathogens [21,22,33], and immune cells [14,17] and manipulates cytokine responses [34,35,36], leading to enhanced pathogen clearance and reduced hyperresponsiveness in the airways and lungs [23]. In this study, we demonstrate that SP-A directly interacts with RV-C15 and inhibits viral attachment and progeny yields in differentiated primary NECs, resulting in the attenuation of antiviral gene and chemokine expression induced by RV-C15 infection. To ensure that the reduced viral copy numbers were not a result of cytotoxic effects of SP-A in NECs, the level of protein synthesis was determined by a leucine incorporation assay (Figure S3a). SP-A did not show any cytopathic effects in the NECs, as shown in Figure 1a.

RV infections mostly occur in the upper airway epithelium, where the immune response to the virus initiates. There has been evidence that immunological defects are present in asthmatic individuals, resulting in suboptimal antiviral responses [37,38,39]. In this study, we compared NECs obtained from non-asthmatic and asthmatic participants. We found that RV-C15 had a higher viral burden in the differentiated NECs from asthmatics. Since the level of cell-associated input virus was higher in the asthmatic group at 4 h p.i. (before virus replication starts), we hypothesized that there may be a higher level of virus receptor expression on the cell surface of the NECs from asthmatics. It has been found that asthma exacerbation risk is associated with the CDHR3 carrying A allele at rs6967330 which changes cysteine 529 to tyrosine (C529- > Y529) [40]. The Y529 variant exhibits higher cell surface expression than C529 in transfected 293T [40] and HeLa [11] cell lines and in differentiated primary airway epithelial cells [10]. Consistent with our hypothesis, 90% of our asthmatic cell donors had an A/A CDHR3 genotype in comparison to ~50% in the non-asthmatic group. It is noteworthy that the inhibition by SP-A was similar in the NECs from the non-asthmatic and asthmatic donors. This indicates that rs6967330 polymorphism or asthma status do not modulate the inhibitory effect of SP-A on RV-C15 infection. As shown in Figure S3b, SP-A treatment did not alter CDHR3 mRNA expression level in NECs.

As expected, SP-A also indirectly attenuates antiviral gene expression and chemokine production induced by RV-C15 infection (Figure 3). This attenuation likely results from the inhibition of viral infection rather than the direct effects of SP-A on antiviral genes or chemokine expression.

There is accumulating evidence that SP-A dysfunction is observed in human asthma [41] and this dysfunction might be related to SP-A2 genetic variations [42,43]. In addition, the level of total SP-A was found to be low in asthma [44,45]. These data demonstrate that asthmatics may be more susceptible to RV-C infection due to a lower quantity and quality of SP-A in the lungs.

The SP-A treatment at 4 h p.i., but not 24 h p.i., reduced viral replication in a dose-dependent manner. These data suggest that early SP-A intervention could attenuate RV-C15 infection by binding to the acquired input virus and reducing the newly produced virus progeny, and reduce its further transmission, but that was not sufficient to neutralize the viruses produced at 24 h. These data suggest that SP-A treatment may further affect virus transmission by reducing the attachment of newly produced RV-C15 progeny to cells during the second replication cycle.

Pulmonary surfactants are composed of approximately 90% lipid and 10% protein by weight, and physiological SP-A concentration is 1.7–2.5 mg/mL [46,47]. Therefore, the highest concentration of SP-A (400 µg/mL) used in this study is physiologically relevant. Kim and coauthors reported that SP-A2, SP-B, and SP-D genes were expressed in the ciliated NECs, but the concentration of SP-A2 protein was not determined [48]. We detected mRNA expression of SP-A2, but not SP-A1, by qPCR in the NEC-ALI cultures from the non-asthmatics. The SP-A protein concentration in the apical wash of these cultures was negligible (less than 250 ng/well). These data suggest that the endogenous level of SP-A protein in differentiated NECs is very low, and it is very unlikely to impact the antiviral effects of exogenous SP-A against RV-C15.

The calcium-dependent interaction strongly suggests that SP-A binds to RV-C15 by the carbohydrate recognition domain (CRD) [23,49], and this interaction interferes with RV-C15’s attachment to the epithelial cells. The detailed mechanisms of SP-A interaction with RV-C15 are still unclear and need further investigation. Even though the cell attachment was reduced by more than 80% by SP-A, a detectable level of RV-C15 binding to the cells was observed. These data suggest that binding of RV-C15 to CDHR3 is not completely blocked by SP-A, or the virus may be able to bind to other yet unknown or non-specific receptor on the cells which is not blocked by SP-A binding.

SP-A binding to solid phase RV-C15 is an empirical observation. However, this binding is dependent on Ca^2+^ and inhibited by EDTA thus following the paradigm of many direct SP-A binding partners. Not all SP-A binding partners are carbohydrates, as the protein binds phospholipids and glycolipids (e.g., DPPC, LPS, and ceramides) [23,49]. The structural basis of the SP-A binding to RV-C is currently unknown. The simplest perspective of SP-A-RV-C15 interactions is that they involve, require, or are attributable to one of the protein’s multiple domains consisting of the following: (1) an N-terminus of the disulfide crosslinks (that participate in covalent oligomerization and enhance the weak interactions of monomers), (2) a collagen-like domain that drives non-covalent trimerization, (3) a pseudo-disordered domain which follows a “knobs in holes reinforcement”, which also promotes/stabilizes oligomerization, and (4) a carbohydrate recognition domain, which exhibits lectin properties (both as monomers and oligomers). The high degree of oligomerization of SP-A (Figure S3) functions to enhance the avidity of its otherwise weak interactions.

To identify amino acid residues in SP-A that play a role in RV-C15 interaction, RV-C15 binding assay using eight variants of rSP-A was performed. The result demonstrated that SP-A2 isoforms, SP-A2 [P_91_, Q_223_], and SP-A2 [A_91_, K_223_], showed high binding affinities to RV-C15 (Figure 5) that were in agreement with viral inhibition. The data demonstrate that amino acids at positions 91 and 223 of SP-A2 may play important roles in RV-C15 binding and provide potential targets for virus–protein interaction studies and antiviral drug design [17,18,20,21,22,36,42,50].

There was a discrepancy in the weak binding of rSP-A2 [A_91_, Q_223_] to RV-C15 and a potency to reduce RV-C15 viral copy numbers by up to 50% (Figure 5 and Figure 6). There is a possibility that the interaction of proteins on a solid phase and in solution could be different due to a conformational change in the protein that is adsorbed on a solid phase. As shown in Figure S3, native SP-A is composed of multiple oligomeric subunits that are resistant to reducing conditions compared to exclusively monomeric rSP-A. Based on a complex multimeric structure of native SP-A, it is difficult to predict the structure of SP-A isoforms based on affinity of the molecules for a given ligand [16,51,52]. The 3D structural analysis of SP-A bound to RV-C capsid may help to investigate the molecular mechanisms of SP-A antiviral properties in the future.

In conclusion, we discovered that SP-A inhibits RV-C15 infection by directly binding to the virus and preventing viral attachment to host cells. The amino acid residues at positions 91 and 223 of SP-A2 might be important determinants of RV-C15 interaction with SP-A and its antiviral properties. These data suggest that SP-A may be a potential therapeutic agent for RV-C infection and RV-C-induced asthma exacerbations.

## Figures and Tables

**Table 1:** Demographic and clinical features of study participants.

	Non-Asthmatic	Asthmatic
**Participants (numbers)**	6	10
**Age (mean (years ± SD))**	15.8 ± 1.1	13.0 ± 2.3
**Sex (Female (%))**	3 (50%)	6 (60%)
**BMI (mean (kg/m^2^) ± SD)**	24.6 ± 6.7	24.1 ± 8.0
**Total IgE (mean (kU/L) ± SD**	21.8 ± 19.7	671.7 ± 793.0
**Eosinophils (mean/µL) ± SD**	1.0 ± 0.4	4.2 ± 2.8
**FeNO (mean (ppb) ± SD**	9.2 ± 3.6	44.4 ± 33.7
**%FEV1 (mean ± SD)**	101.9 ± 7.4	90.8 ± 19.8
**CASI score (mean ± SD)**	0.0 ± 0.0	5.6 ± 2.3
**CDHR3 Genotype (A/A, G/A)**	A/A = 3 G/A = 2 Undetermined = 1	A/A = 9 G/A = 1

## Data Availability

Data are available upon request.

## Supplementary Materials

The following supporting information can be downloaded at: https://www.mdpi.com/article/10.3390/v16111709/s1, Table S1. Primer sequences for RT-qPCR assay; Figure S1. SP-A showed low level of non-specific attachment to the microtiter plate. Figure S2. Native SP-A contains molecular structure that is more resistant to reducing agent than recombinant SP-A1 [V_50_, R219]. Figure S3. SP-A does not reduce metabolism of differentiated NECs and no impact on CDHR3 expression of differentiated NECs in ALI cultures.

## Abbreviations

SP-A	Native Surfactant protein A
RV	Rhinovirus
RV-C15	Rhinovirus C15
RV-C15-GFP	Rhinovirus C15 expressing green fluorescent protein (GFP)
p.i.	Post infection
NECs	Nasal epithelial cells
ALI	Air-liquid interface
CDHR3	Cadherin-related family member 3
DPBS	Dulbecco’s phosphate-buffered saline
MDA5	Melanoma differentiation-associated protein 5
IRF7	Interferon regulatory factor 7
IFNλ	Interferon lambda
CXCL11	C-X-C motif chemokine 11
FFU	Fluorescent focus forming units
RT-qPCR	Quantitative reverse transcription polymerase chain reaction
A_490nm_	Absorbance value at 490 nanometers
