# Supplementary material for: Surfactant Protein A Inhibits Human Rhinovirus C Binding and Infection of Airway Epithelial Cells from Pediatric Asthma
Source: Viruses. 2024 Oct 30;16(11):1709. doi: 10.3390/v16111709 (PMC11598966; doi:10.3390/v16111709)
Supplement: Supplementary file 1 [file viruses-16-01709-s001.zip › viruses-3158577-supplementary.pdf]

Supplemental table and figures

Table S1. **Primer sequences for RT-qPCR assay**

**Primer/ probe**	**Sequence (5'-3')/ catalog number**
beta Actin F	CACTCTTCCAGCCTTCCTTC
beta Actin R	GTACAGGTCTTTGCGGATGT
RVC15 F	CCTCCGGCCCCTGAAT
RVC15 R	AAACACGGACACCCAAAGTAGT
IFNλ F	GCCAAAGATGCCTTAGAAGAG
IFNλ R	CAGAACCTTCAGCGTCAGG
MDA5 F	GAAGTACAATGAGGCCCTACAA
MDA5 R	CATCACCACCCTCATCACTATC
IRF-7 F	ACACACACATGCTGGACTC
IRF-7 R	CTTGGTTGGGACTGGATCTG
CXCL11 probe	Hs00171138_m1
GUSB probe	Hs.PT.51.2648420
CDHR3 F	TGTGAAGGATGAGGTTGGTG
CDHR3 R	TCCAGGGTTTGCTCTTTCTAC

F, forward; R, reverse.

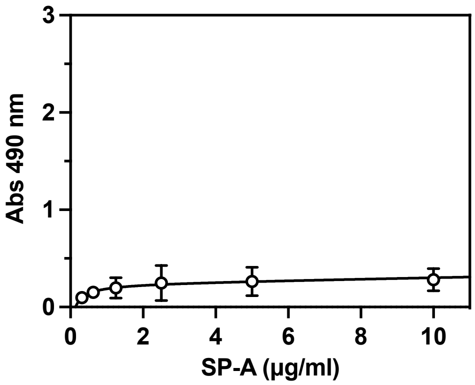

Figure S1. SP-A showed low level of non-specific attachment to the microtiter plate.

SP-A (0 to 10 µg/ml) was incubated for 1hr in PBS-precoated microtiter plate (without RV-C15). The binding of SP-A to the plate was quantified by anti-human SP-A HRP-conjugated antibody in the presence of 5 mM $CaCl_2$. The results represent means ± SD from 3 independent experiments.

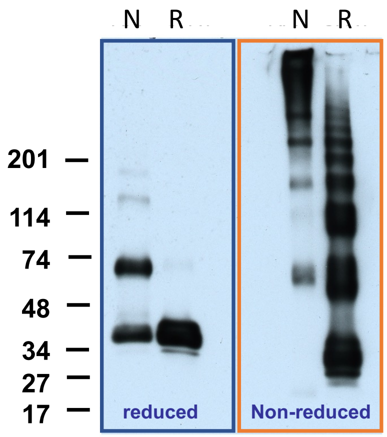

Figure S2. The structure of native SP-A is more resistant to reducing agent than that of recombinant SP-A1 [V_{50}, R_{219}].

Native (N) SP-A and recombinant (R) SP-A1 [V_{50}, R_{219}] were analyzed by SDS-PAGE under reducing (with β-mercaptoethanol) and non-reducing (without β-mercaptoethanol) conditions. The samples were subjected to heating at 95°C for 5 min and electrophoresis on 4 –20% acrylamide gradient gels at 100 V for 1 h. The molecular weight marker is shown on the left.

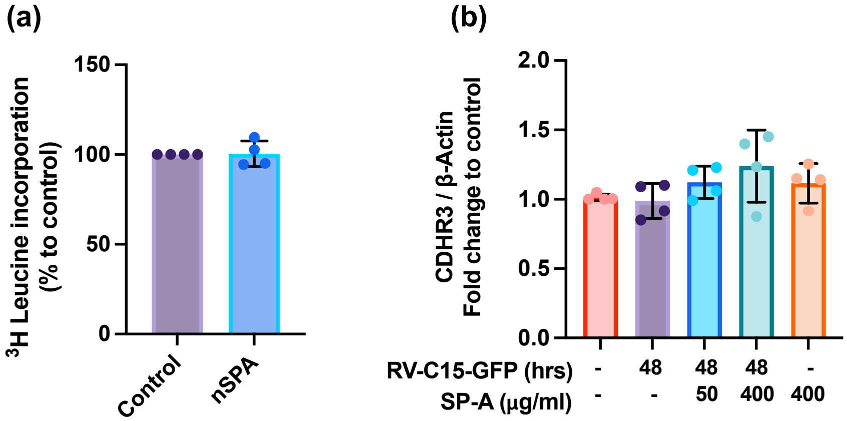

Figure S3. SP-A does not reduce metabolism and does not affect CDHR3 expression in differentiated NEC-ALI cultures.

(a) Differentiated NECs were incubated with ^{3}H-leucine containing media in the presence or absence of SP-A at 400 µg/ml for 48 hr. Newly synthesized ^{3}H-containing protein was quantified by liquid scintillation spectrometry. The data are shown as means ± SD. The control was set up as 100% (b) CDHR3 mRNA levels were measured by RT-qPCR in each condition (control (no virus or SP-A), RV-C15-GFP alone, RV-C15-GFP + SP-A at 50µg/ml, RV-C + SP-A at 400µg/ml, SP-A alone, 400µg/ml, at 48hrs). We used the primers shown in Table 1. The experiments were performed in duplicate using differentiated NECs from 4 different donors. SP-A treatment did not alter CDHR3 mRNA expression in NECs by the analysis with two way Anova and Turkey's multiple comparison analysis.
